# Obstetrics care providers attitude and utilization of non-pharmacological labor pain management in Harari regional state health facilities, Ethiopia

**DOI:** 10.1186/s12884-022-04717-9

**Published:** 2022-05-04

**Authors:** Addis Eyeberu, Adera Debela, Tamirat Getachew, Merga Dheresa, Addisu Alemu, Yadeta Dessie

**Affiliations:** 1 School of Nursing and Midwifery, College of Health and Medical Sciences, Haramaya University, Harar, Ethiopia; 2 School of Public Health, College of Health and Medical Sciences, Haramaya University, Harar, Ethiopia

**Keywords:** Attitude, Utilization, Non-pharmacological labor pain relief methods, Labor pain, Harar

## Abstract

**Background:**

In a woman’s life, labor pain is the most severe pain that they have ever faced. In Ethiopia, the provision of pain relief in labor is often neglected. Furthermore, evidence strongly urged that further research is needed on non-pharmacological labor pain management. Therefore, obstetrics care providers’ attitudes and utilization of non-pharmacological labor pain management need to be assessed.

**Method:**

A facility-based cross-sectional study was conducted from May 20 to June 10, 2021, in Harari regional state health facilities, Ethiopia. All obstetric caregivers in Harari regional state health facilities were included in the study. A structured questionnaire adapted from the previous studies was used to collect data. The data was entered into Epi-data version 3.1 statistical software. Statistical analysis was carried out by using SPSS for windows version 22. Multivariate linear regression analysis was employed to determine the association between independent variables and the outcome variable.

**Result:**

The overall utilization of non-pharmacological labor pain relief methods was 59.3% [(95% CI (53.9,63.4)]. Three hundred five (65.5%) of the study participants had unfavorable attitudes. Females compared to males (β = − 0.420; 95% CI: − 0.667, − 0.173), clinical experience (β = − 0.201; 95% CI: − 0.268, − 0.134), knowledge sum score (β =0.227: 95%; CI: 0.18,0.247), and attitude sum score (β = 0.376; 95% CI: 0.283, 0.47) were showed significantly association with utilization of non-pharmacological labor pain management.

**Conclusion:**

The overall utilization of non-pharmacological labor pain relief methods was relatively good compared to other studies done in Ethiopia but all women’s need for labor relief methods should not be ignored. In this study sex of the respondents, clinical experience, individual preference, attitude and knowledge were factors associated with the utilization of non-pharmacological labor pain management. All stake holds need to work together to improve the attitude of health providers and to increase the utilization of non-pharmacologic labor pain management.

**Supplementary Information:**

The online version contains supplementary material available at 10.1186/s12884-022-04717-9.

## Background

Labor is characterized by regular, painful uterine contractions that increase in frequency and intensity in three stages of labor. The extent to which a woman feels in control of pain during labor is an indicator of maternal emotional wellbeing in childbirth [1]. Labor pain management is generally categorized into two, pharmacologic and non-pharmacologic labor pain management. Nonpharmacological options include emotional support, directed breathing and relaxation techniques, massage, laboring in water, and the use of transcutaneous electrical nerve stimulation (TENS) [2]. The pain a woman experiences during labor and birth is individualized and caused by several factors [3]. Mother with intense labor pain affected by emotional distress, depression, and anxiety. Therefore, pain control during labor is needed to reduce such consequences.

Evidence indicated that most women signified that they were able to cope with labor pain through non-pharmacological management and they were highly satisfied with non-pharmacological management [4, 5]. Similar evidence also showed that utilization of non-pharmacologic labor pain management averts the adverse effect of pharmacologic pain relief methods [6].

In Ethiopia, the overall utilization of non-pharmacologic labor pain management was between 37.9 and 46.8% [7–9]. Non-pharmacological labor pain methods were proved effective in alleviating pain during the first and second stages of labor [5, 10]. Inadequate knowledge, negative attitudes, lack of trained personnel, and absence of protocols were barriers to the use of pain relief methods [9, 11]. There were significant associations between respondents’ educational level and rubbing of the back/ massage, position change, cold/warm bath, relaxation, and social support [12].

A study done in Kenya [13] and Ethiopia [7] showed that 56 and 47.6% of participants knew labor pain relief methods respectively. However, nearly half percent, (49.1%), of the participants responded that nonpharmacological methods of pain relief can produce harmful effects on newborns [7]. Another evidence also showed that 48.5% of obstetrics care providers know non-pharmacologic labor pain management methods [14]. The knowledge level is one of the determinant factors for the provision of non-pharmacologic methods.

Evidence showed that 65.3% of obstetrics care providers had a negative attitude toward obstetric labor pain relief methods [7]. Which has a greater impact on the provision of non-pharmacologic methods to the laboring mothers. A study done in the Tigray region showed that obstetrics care providers who have a positive attitude toward managing labor pain were more likely to use labor pain management methods than those who have a negative attitude [14]. The attitude of obstetrics care providers affects the care provided to the laboring mothers and their need to relived the pain is diminished by the poor attitude. Evidence showed that there were concerns about the way and manner some health care providers approach and communicate with patients in health facilities [15].

Even though the Federal Ministry of Health (FMOH) drafted Ethiopian Emergency Obstetric and Newborn Care (EmONC) assessment standards to enhance appropriate management of labor pain and management of complications during labor and delivery [16]. There is no specific protocol that aids OCPs to update themselves and equip with the necessary knowledge and attitude.

The provision of pain relief in labor is often neglected. Most women in Ethiopia do not receive adequate pain relief. Obstetrics care providers (OCPs) have an important role in providing pain relief options during labor. In the major of obstetrics care providers, the pharmacological pain relief method is favored as the only method known to us. Lack of emotional support and excessive medical intervention for laboring women result in increased intensity of pain. Furthermore, evidence strongly urged that further research is needed on non-pharmacological labor pain management [17]. Therefore, healthcare providers’ attitudes and utilization of non-pharmacological labor pain management need to be assessed.

### Research questions

What is the overall utilization of non-pharmacological labor pain management among obstetric care providers?

What is the level of obstetric care providers’ attitude towards non-pharmacological labor pain management?

What are the factors associated with the utilization of non-pharmacological labor pain management?

## Methods

### Study design, study setting, and period

A facility-based cross-sectional study was conducted in Harari regional state health facilities in Ethiopia from May 20 to June 10, 2021. The region is located 526 km from Ethiopia’s capital city, Addis Abeba. Oromia is a regional state that encircles the Harari region. The Harari region is divided into nine woredas, three of them are rural and the other six are urban. The urban districts are divided into 19 kebeles (The smallest administrative units), while the rural districts are divided into 17 peasant associations (which is equivalent to kebeles in urban cases). The region’s total population (2021 projection based on the 2007 Census, CSA) is 270,000, with 136,000 males and 134,000 females (1). There are 59,487 total households [18]. In the region, there are two public hospitals, two private hospitals, two governmental hospitals, eight public health centers, and twenty health posts.

### Study population

The study population consisted of all obstetric care providers working in Harari regional state health facilities during the study period. Because the source population in the study area was small, all obstetric caregivers who were available during the study period were included. Obstetrics care providers who were unable to collect data due to illness, annual leave, or training were excluded from the study.

### Sample size determination and sampling procedure

The study was conducted at maternal health facilities including two public hospitals (HFSUH and Jugol hospital), one private hospital (Harar General hospital), two private clinics, and nine Harari regional state health centers. Because the number of OCPs in all health facilities was insufficient, the study included all obstetrics care providers (464).

### Data collection methods and data quality control

A structured questionnaire was prepared by adapting from different studies [8, 9, 19]. The questionnaire developed for this study is provided as Additional File 1. The questionnaire included four essential components: socio-demographic characteristics of providers, knowledge, attitude, and utilization-related questions. Forty-two questions were included in the questionnaire’s four components. Before beginning the study, the adapted questionaries were validated. To adapt the questionaries, the first step was to review different types of similar literature. The second stage involved ensuring that the adapted questionaries were cross-validated. The tool was given to a group of English-speaking respondents, and their responses were compared to detect differences in incomprehension. Pre-testing was the final step. Before the study began, a pretest was administered to 25 obstetric care providers at Dil Chora referral hospital in Dire Dawa city administration. The questionaries were modified based on the pretest findings, such as typos and question suitability. The instrument’s validity and reliability were then evaluated. Cronbach’s Alpha was used to assess the quality of the instruments we used. The final score was 0.79. Following ethical approval, a letter of permission was written to all health facilities to allow us to proceed with the study. After obtaining permission, the data collectors provided a detailed explanation of the study’s purpose and the significance of their involvement with the obstetrics care providers. Respondents who volunteered to complete self-administered and pre-tested questionnaires were included. The questionnaires were given one-on-one. The information was gathered from all of the region’s healthcare facilities. The data were collected by five well-trained BSC-holding Nurses and supervised by two MSC-holding Midwives.

The collected data were checked for completeness and consistency on a daily basis. Supervisors and data collectors were also trained.

### Variables

#### Outcome variable

Utilization of non-pharmacologic pain relief methods.

#### Independent variables

Socio-demographic variables, knowledge of OCPs towards non-pharmacologic pain relief methods, Attitudes of obstetrics care providers (OCPs) towards non-pharmacologic pain relief methods, and institutional related variables.

### Measurement

#### Non-pharmacological labor pain management methods

OCPs who used methods that were greater than or equal to the mean value of utilization-related questions [8, 9]. Twelve items were used to assess the use of non-pharmacological labor pain management methods. The questions were answered with yes or no/I don’t recall responses. Those who said “Yes” were coded with a “1,” while those who said “No/I don’t remember” were coded with a “0.” The total score was then computed. The lowest and highest possible scores were 0 and 9, respectively.

#### Obstetric caregivers

Midwifery, Nurses, Health Officers, and residents.

#### Knowledge

A total of 15 items were used to assess knowledge of non-pharmacological labor pain management methods. The questions were answered with yes, no, or I don’t know responses. During analysis, the correct answer was coded with “1” and the incorrect, or I don’t know, the answer was coded with “0.” The sum score (range 0–15) was then computed and categorized.

#### Adequate knowledge

Was defined as OCPs who answered more than or equal to the mean values (6.65) of knowledge-related labor pain relief method questions [8].

#### Inadequate knowledge

OCPs who answered less than the mean value (< 6.649) of knowledge-related questions were considered to have inadequate knowledge about labor pain relief methods [8].

#### Favorable attitude

OCPs who answered questions about attitude-related labor pain relief methods with a value greater than or equal to the mean value (≥ 7.5) were considered to have favorable attitudes toward labor pain relief methods [7].

Unfavorable attitude: OCPs who answered less than the mean value (< 7.5) of the total attitude-related questions were classified as having an unfavorable attitude toward labor pain relief methods [7].

### Statical analysis

The data was coded, cleaned, and edited before being entered into Epi data statistical software version 3.1 and exported to SPSS window version 22 for analysis. The characteristics of study participants, such as socio-demographics, level of knowledge, and attitude of obstetrics care providers, were described using descriptive statistical analysis, such as simple frequency. The data was then presented in the form of frequencies, tables, and figures. Linear regression analysis was used to identify factors associated with the use of non-pharmacological labor pain management by treating the cumulative score of use of non-pharmacological labor pain management as a continuous variable. The variables were selected using bivariate analysis and dropped from further inclusion in the multivariate model if their *p*-values were greater than 0.25. Finally, the multivariable linear regression model was fitted using a backward stepwise elimination method, and variables with *p*-values less than 0.05 in the final model were considered significant predictors of non-pharmacological labor pain management utilization. The standardized with % CI was used to measure the amount by which the dependent variable changes if the independent variable is changed by one unit while the other independent variables remain constant. The Hosmer-Lemeshow statistic and omnibus tests were used to assess the goodness of fit. The multi co-linearity test was used to examine the correlation between independent variables using standard error and collinearity statistics (variance inflation factors greater than 10 and standard error greater than 2 were considered suggestive of the presence of multi co-linearity). *P*-value0.05 was used in this study to declare a result statistically significant.

### Ethical considerations

Haramaya University’s College of Health and Medical Sciences’ Institutional Health Research Ethics Review Committee provided ethical approval for this study (HU-IHRERC). As needed, official letters to conduct a study were obtained from health facilities. The purpose of the study, as well as the risks and benefits of the study, were explained to the health facilities. Before data collection, informed, voluntary, written, and signed consent was obtained from health facility administrators and study participants.

## Results

### Socio-demographic characteristics

A total of 464 health care professionals participated in this study. Among them 200 (43.1%) were males and 264 (56.9%) were females which is a 0.76 to 1 male to female ratio. More than half 233(53%) of the study participants were Midwives. More than half 284 (61.2%) of obstetric care providers were Bachler of Sciences in their level of education (Table 1).

**Table 1:** Socio-demographic characteristics of obstetric care providers at Harari regional state public health facilities, 2021 (*n* = 464)

Variables	Categories	Frequency	Percentage
Sex	Male	200	43.1
	Female	264	56.9
Age	15–24	60	12.9
	25–54	404	87.1
Religion	Orthodox	220	47.4
	Muslim	176	37.9
	Protestant	52	11.2
	Others	16	3.4
Profession	Midwife	233	53
	Nurse	198	42.67
	Obstetrician	20	4.3
	Health officers	13	2.8
Level of education	Diploma	142	30.6
	BSC	284	61.2
	MSC	38	8.2
Clinical experience	< 2 years	88	19
	≥2 years	376	81

*others = Catholic and Adventist

### Knowledge and attitudes of obstetric care providers regarding non-pharmacologic Labor pain management

Almost all 444 (95.7%) health care providers know about labor pain relief methods. Among them, 384(82.76%) know about non-pharmacological pain relief methods and other methods while 56 (12.6%) know only about non-pharmacological pain relief methods. Among non-pharmacological methods, 240 (62.5%) of obstetric care providers know about psychotherapy while 156 (40.6%) of obstetric care providers know about massaging back of the mothers. More than half 252 (54.3%) of obstetric care providers know about the WHO pain ladder. The mean score of knowledge questions is 6.65(SD = 2.95) with minimum and maximum scores of 0 and 15 respectively. Almost half 230 (49.6%) of the respondent have adequate knowledge (Table 2).

**Table 2:** knowledge and attitudes of obstetrics care providers about non-pharmacological pain relief methods in Harari regional state public health facilities, 2021

Variable	Categories	Frequency	Percentage
Do you know about labor pain management methods (*n* = 464)	Yes	444	95.7
	No	20	4.3
Type of labour pain management you know (*n* = 444)	Only non-pharmacology	56	12.1
	Only pharmacology	80	17.2
	Both	328	73.9
From non-pharmacology methods, which methods do you know (*n* = 384)			
Do you know psychotherapy	Yes	240	62.5
	No	144	37.5
Do you know to allow the mother to ambulate	Yes	100	26
	No	284	74
Do you know massage the back	Yes	156	40.6
	No	228	59.4
Do you know allow vertical positioning	Yes	132	34.4
	No	252	65.6
Do you know show how to bear down	Yes	48	12.5
	No	336	87.5
Do you know allow her choice	Yes	80	20.8
	No	304	79.2
Do you know hot compress	Yes	32	8.3
	No	352	91.7
Do you know music therapy	Yes	96	24.7
	No	292	75.3
Which method is your individual preference	Non-pharmacologic	136	29.3
	Pharmacologic	52	11.2
	Both	276	59.5
Do you know WHO pain ladder	Yes	252	54.3
	No	212	45.7
Pain expectation	Mild	36	7.8
	Moderate/sever	428	92.2
Training	Yes	296	63.8
	No	168	36.2
Availability of protocols	Yes	417	89.9
	No	47	10.1
Believe that the labour pain management methods help the mother to cope with labour pain (*n* = 464)	Yes	392	84.5
	No	72	15.5
Do you think every mother during labour should be managed (*n* = 464)	Yes	320	69
	No	144	31
Believe that even though labor pain is natural and the mother hasn’t to face it (*n* = 464)	Yes	344	74.1
	No	120	25.9
Think non-pharmacology methods are necessary for managing labour pain	Yes	280	60.3
	No	184	39.7
Believe that you have a responsibility and obligation to manage labour pain?	Yes	392	84.5
	No	72	15.5

Regarding attitudes of obstetrics care providers, 392 (84.5%) of the obstetric care providers believe that labor pain management methods can alleviate or help the mother to cope with labor pain. Three hundred twenty (69%) of obstetric care providers thinks that mother during labor should be managed and 344 (74.1%) of obstetric care providers believe that even though labor pain is natural, mothers should not face it. More than half of 280 (60.3%) of OCPs believe that analgesics are necessary to manage labor pain. More than two-thirds (84.5%) of OCPs believe that they have the responsibility and obligation to manage labor pain. The mean score of the attitude is 4.06 (SD = 1.4) with minimum and maximum scores of 6 and 12 respectively. Three hundred five (65.5%) of the study participants have unfavorable attitudes (Table 2).

### Utilization of non-pharmacologic Labor pain management

Of the total of 464 obstetric care providers, 364 (78.4%) obstetric care providers utilized pain relief methods for laboring mothers. Among those, 128 (27.6%) of OCPs provide only non-pharmacological pain relief methods. The most commonly provided methods were psychotherapy 260 (78.3%), massage of the back 228 (68.7%), and allowing the mother to ambulate 154 (55.4%). The main reasons for the non-use of pain relief methods included high patient flow 172 (54.4%), the limited number of staff 164 (51.9%), lack of knowledge and skill 104 (32.9%). (Table 3).

**Table 3:** Non-pharmacologic labor pain relief methods provided at Harari regional state public health facilities, 2021 (*n* = 464)

Variable	Categories	Frequency	Percentage
Labor pain relief methods provided in the past month (*n* = 464)	Yes	364	78.4
	No	100	21.6
Pain relief methods utilized (*n* = 364)	Only non-pharmacologic	128	35.2
	Non-pharmacologic with others	204	56
	Only pharmacologic	32	8.8
Utilized non- pharmacologic labor pain methods (*n* = 332)			
Psychotherapy	Yes	260	78.3
	No	72	21.7
Allow the mother to ambulate	Yes	184	55.4
	No	148	44.6
Massage the back	Yes	228	68.7
	No	104	31.3
Allow vertical positioning	Yes	40	12
	No	292	88
Show how to bear down	Yes	40	12
	No	292	88
Allow her choice	Yes	20	6
	No	312	94
Hot compress	Yes	24	7.2
	No	308	92.8
Music therapy	Yes	56	16.9
	No	276	83.1

The mean score of utilization non-pharmacological labor pain management was 2.61(SD = 1.64) with minimum and maximum scores of 0 and 9 respectively. About 275 (59.3%) of the study participants utilized non-pharmacological labor pain management.

### Factors associated with utilization of non-pharmacological Labor pain managements

Variables like age, sex, clinical experience, caregiver preferences, level of education, pain expectation of caregiver, availability of protocols, level of knowledge, and attitude of the respondents towards non-pharmacologic labor pain management were associated with simple linear regression analysis.

In the final multiple linear regression model sex of the respondents, clinical experience, pain expectation, individual preference, attitude, and knowledge of non-pharmacological labor pain management were significantly associated with utilization of non-pharmacological labor pain management (*p*-value < 0.05). As the clinical experience increase by 1 year, the utilization of non-pharmacological labor pain management score will increase by − 0.201 if the effects of other variables keep constant (β = − 0.201; 95% CI: *−* 0.268, − 0.134). Females compared to males (β = − 0.420; 95% CI: − 0.667, − 0.173) and respondents having sever pain expectation (β: -0.367; 95% CI: *−* 0.539, − 0.116) showed significantly association with utilization of non-pharmacological labor pain management. For a unit increase in the knowledge score, the utilization of non-pharmacological labor pain management will increase by 0.227 if the effects of other factors keep constant (β =0.227: 95%; CI: 0.18,0.247). Furthermore, if the score of attitudes increased by one unit, the utilization of non-pharmacological labor pain management will increase by 0.376 keeping other factors constant (β = 0.376; 95% CI: 0.283, 0.47) (Table 4).

**Table 4:** Multiple linear regression analysis of factors associated with utilization of non-pharmacological labor pain management methods among obstetric caregivers at Harari regional state public health facilities, 2021 (*n* = 464)

Variables	Unstandardized Coefficients		Standardized Coefficients	Sig.	95% CI, B
	B	Std. Error	Beta		
Sex (Female Vs Male)	−0.420	0.126	−0.127	0.001	−0.667, −0.173
Age in years	0.022	0.024	0.051	0.357	− 0.025, 0.68
Level of education (diploma Vs BSc and above)	0.230	0.139	0.066	0.098	−0.043, 0.504
Clinical experience in years	−0.201	0.034	−0.299	0.001	−0.268, − 0.134
Pain Expectation (Severe Vs Mild/ Moderate)	−0.327	0.107	−0.120	0.002	−0.539, − 0.116
Training (yes Vs no)	− 0.216	0.133	− 0.063	0.105	− 0.477, 0.45
Individual preference (non-pharmacologic Vs pharmacologic)	0.415	0.091	0.175	0.001	0.237, 0.593
Knowledge of non-pharmacological methods (sum score)	0.227	0.024	0.409	0.001	0.18, 0.247
Attitude towards non-pharmacological methods (sum score)	0.376	0.048	0.318	0.001	0.283, 0.47

## Discussion

Evidence suggested that obstetrics care providers’ attitudes and utilization of non-pharmacologic labor pain management were not studied well. Therefore, this study was done to assess obstetrics care providers’ attitudes and utilization of non-pharmacological labor pain management methods in Harari regional state health facilities. Additionally, factors that affect the utilization of non-pharmacological labor pain management methods were isolated.

The overall utilization of non-pharmacological labor pain relief methods was 59.3% [(95% CI (53.9,63.4)]. The finding is higher than studies done in general hospitals of Tigray 43.3% [14], East Gojjam zone, Amhara region 30.4% [19], Gondar 20.72% [20], Amhara region,40.1 and 46.8% [8, 9]. The possible explanation could be variations in the level of knowledge, the higher sample size of the current study, and the availability of labor-management protocols which enhance the utilization of non-pharmacological pain management methods.

The finding of this study was lower than the study conducted in Ethiopia which was 66% [21], This discrepancy might be due to the sample size difference, study period difference, the health facility setups because some of the setups could be well-equipped, the presence of skilled man powers (who took training may provide pain relief to laboring mothers). Even if the overall utilization of non-pharmacologic labor pain management was higher than the majority of studies done in Ethiopia, women’s need for labor pain relief methods should not be ignored. To increase the utilization of non-pharmacologic labor pain management among OCPs, facilitating training, increasing the number of OCPs, and improving the knowledge of OCPs should be soughed seriously.

In this study, three hundred five 65.5% [(95% CI (60.9, 69.8)] of the study participants have unfavorable attitudes. This is higher than studies done in Benishangul Gumuz Regional State 53% [22] and Gondar Ethiopia 58.04% [20]. This discrepancy may be due to socio-cultural differences among study participants and a higher sample size of the current study than studies done in Gondar [20] and Benishangul Gumuz Regional State [22]. This implied that concerned bodies should work together to improve the attitude of obstetrics care providers through different strategies such as facilitating continuous educational opportunities which have a profound effect on addressing the women’s need of alleviating labor pain. Obstetrics care providers might have several reasons for their unfavorable attitudes, so identifying those reasons through other qualitative studies might be a beginning for finding a solution to this problem.

This study identified that the sex of the respondents, clinical experience, pain expectation, individual preference, attitude, and knowledge of non-pharmacological labor pain management were significantly associated with the utilization of non-pharmacological labor pain management.

In this study, being female was associated with the utilization of non-pharmacological labor pain management. The possible justification is that females face the pain as they go through the childbirth process and know the severity of pain and the need for pain relief methods then which increases the utilization of non-pharmacological labor pain management.

In this study, OCPs’ pain expectations and individual preferences were associated with the utilization of non-pharmacological labor pain management. The possible justification is that those OCPs expecting labor pain as severe would understand the need for pain relief methods and can utilize non-pharmacologic pain relief methods. Similarly, those OCPs preferring non-pharmacologic labor pain management would utilize it.

In this study, clinical experience was associated with the utilization of non-pharmacological labor pain management. Evidence showed that OCPs with clinical experience influence their attitudes about and intent to provide labor pain management then which increases the utilization of non-pharmacological labor pain management [23]. The possible justification is that those study participants serving for many years had sufficient knowledge and skill which in turn increased the utilization of non-pharmacologic labor pain management.

In this study, if the score of attitudes increased by one unit, the utilization of non-pharmacological labor pain management will increase by 0.376 keeping other factors constant. Evidence showed that the attitude of OCPs was significantly associated with the utilization of pain relief methods and it is recommended to improve the attitude of OCPs to scale up the utilization of pain relief methods [11].

Knowledge of obstetrics care providers was another factor associated with the utilization of non-pharmacologic labor pain management. In this study, for a unit increase in the knowledge score, the utilization of non-pharmacological labor pain management will increase by 0.227 if the effects of other factors were kept constant. This could be explained as an increase in knowledge and awareness level will make the obstetrics care providers utilize the pain relief method.

The strength of this study is the use of a larger sample size. Another strength of this study is it provides information on the overall utilization of non-pharmacologic labor pain management methods that can help health facilities in the Harari regional state to plan for appropriate intervention and to improve non-pharmacologic labor pain management methods by identifying the gap to take a corrective measure. The more information health providers and administrative bodies have about the practice of non-pharmacologic labor pain management methods in the study area, the more they understand the problem and set strategies.

The limitation of this study was it might not indicate a cause-effect relationship because the study design was cross-sectional. Since the study is institution-based and didn’t incorporate women in labor, the results might lack generalization to the entire population in the catchment area.

## Conclusions

The overall utilization of non-pharmacological labor pain management methods was relatively good compared to other studies done in Ethiopia but all women’s need for labor relief methods should not be ignored. This study also identified that nearly three fourth of the respondents had unfavorable attitudes. In this study sex of the respondents, clinical experience, individual preference, attitude and knowledge were factors associated with the utilization of non-pharmacological labor pain management. All stake holds need to work together to improve the attitude of health providers and to increase the utilization of non-pharmacologic labor pain management. To increase the utilization of non-pharmacologic labor pain management among OCPs, facilitating training, increasing the number of OCPs, and improving the knowledge of OCPs should be soughed seriously.

## Supplementary Information

**Additional file 1.** Questionaries developed for this study.

## Data Availability

All relevant data are included in this study. However, additional data will be available from the corresponding author on reasonable request.

## Abbreviations

AOR: Adjusted Odds Ration
EmONC: Emergency Obstetric and Newborn Care
LMIC: Low Mid Income Country
OCP: Obstetrics Care Providers
WHO: World Health Organization
