# Supplementary material for: Obstetrics care providers attitude and utilization of non-pharmacological labor pain management in Harari regional state health facilities, Ethiopia
Source: BMC Pregnancy Childbirth. 2022 May 4;22:389. doi: 10.1186/s12884-022-04717-9 (PMC9066716; doi:10.1186/s12884-022-04717-9)
Supplement: Supplementary file 1 — Additional file 1. Questionaries developed for this study. [file 12884_2022_4717_MOESM1_ESM.docx]

- 1. Questionnaire

Circle or write the appropriate response

Part I socio demographic

| S.  No | Question | Response | Skip |
| --- | --- | --- | --- |
| 101 | Age in year? | _____________ |  |
| 102 | Gender? | 1. Male  2. female |  |
| 103 | Religion? | 1. Orthodox 2. Muslim 3. Protestant 4. Other 5. Other specify____ |  |
| 104 | Profession? | 1. Health officer 2. Midwife 3. Nurse |  |
| 105 | Level of education? | 1. Diploma 2. BSc 3. MSC |  |
| 106 | Clinical experience in year? | _____________ |  |
| PART II Knowledge related questions | | | |
| 201 | Do you know about labour pain management methods? | 1. Yes 2. No | 207 |
| 202 | If “yes” to question No 201, what type of labour pain management do you know? | 1. Pharmacologic 2. Non-pharmacologic 3. Both 4. Un-sure |  |
| 203 | If “Pharmacological” to question No 202, which pharmacologic method do you know? More than one answer is possible | 1. Systemic opioids 2. NASID drugs 3. Epidural analgesia 4. Inhalational 5. If other specify_______. |  |
|  | If “non- pharmacological” to question No 202, which type of non-pharmacologic method, do you know? More than one answer is possible…. | | |
| 204 | Psychotherapy | 1. Yes 2. No |  |
| 205 | Allow the mother to Ambulate | 1. Yes 2. No |  |
| 206 | Massage the back | 1. Yes 2. No |  |
| 207 | Allow free vertical positioning | 1. Yes  2. No |  |
| 208 | Transcutaneous electrical nerve stimulation | 1. Yes  2. No |  |
| 209 | Show the patient how to bear down | 1. Yes  2. No |  |
| 210 | Acupuncture | 1. Yes  2. No |  |
| 211 | Hypnosis | 1. Yes  2. No |  |
| 212 | Allow companion of her Choice | 1. Yes  2. No |  |
| 213 | Music therapy | 1. Yes 2. No |  |
| 214 | If other specify______. | ------------ |  |
| 215 | If you know about labour pain relief methods, do they have side effect on labour and delivery outcome? | 1. Yes 2. No 3. I don’t know |  |
| 216 | If” yes” to question 205, what is the side effect on labour and its outcome? More than one answer is possible | 1. Delay progress of labour 2. Cause fetal distress 3. Increase instrumental Delivery 4. Increase C/S delivery 5. If other specify______. |  |
| 217 | Have you asked laboring women to provide labour pain relief method? | 1. Yes 2. No 3. I don’t remember |  |
| 218 | Have you heard about WHO pain ladder? | 1. Yes 2. No 3. Unsure |  |
| III. Attitude related questions | | | |
| 301 | Do you believe labour pain management methods can alleviate or help the mother to cope labour pain? | 1. Yes 2. No |  |
| 302 | Do you think every mother during labour should be managed? | 1. Yes 2. No |  |
| 303 | Do you believe that even though labour pain is natural and mother hasn’t to face it? | 1. Yes 2. No |  |
| 304 | Do you think analgesic is necessary for managing labour pain? | 1. Yes 2. No |  |
| 305 | Do you believe that you have responsibility and obligation to manage labour pain? | 1. Yes 2. No |  |
| 306 | Would you provide labour pain relief methods if you had resources? | 1. yes 2. No 3. Unsure |  |

| PART IV Practice Related Questions | | | |
| --- | --- | --- | --- |
| 401 | Have you ever provided any labour pain relief method in the past one month? | 1. Yes 2. No 3. Don’t remember |  |
| 402 | If” yes” to question No 401, which method? | 1. Pharmacological 2. Non-pharmacological 3. Both |  |
| 403 | If “Pharmacological” for question403 which method? You can answer more than one…. | 1. Pethidine 2. Diclofenac 3. Paracetamol 4. Hyoscine 5. If other specify_______. |  |
|  | If “non-pharmacological” to question 403, which type non pharmacologic you can answer more than one... | | |
| 404 | Have you provided psychotherapy | 1. Yes 2. No |  |
| 205 | Have you provided allow the mother to Ambulate | 1. Yes 2. No |  |
| 206 | Have you provided massage the back | 1. Yes 2. No |  |
| 207 | Have you provided allow free vertical Positioning | 1. Yes 2. No |  |
| 208 | Have you provided show the patient how to bear down | 1. Yes 2. No |  |
| 209 | Have you provided allow companion of her Choice | 1. Yes 2. No |  |
| 210 | Have you provided hot compress | 1. Yes 2. No |  |
| 211 | Have you provided music therapy | 1. Yes 2. No |  |
| 212 | If other specify_______. | 1. Yes 2. No |  |

| V. Personal preference &pain expectation question | | | |
| --- | --- | --- | --- |
| 501 | Which method would you prefer for managing labour pain? | 1. Pharmacologic 2. Non-pharmacologic 3. Both |  |
| 502 | What is your expectation of labour pain? | 1. Mild pain 2. Moderate pain 3. Severe pain |  |
| VI. Questions on reasons for non-use of labour pain management methods | | | |
| 601 | Reasons for non-utilization of labour pain management methods? Multiple options are possible…. | 1. High patient flow 2. Lack of knowledge & Skill 3. No drug 4. No equipment 5. Small number of staff 6. If other specify_______ |  |
| VII. Questions related to institutional factors | | | |
| 701 | If you know pharmacologic methods, is their labour pain managing drugs/ analgesics available at your hospital? | 1. Yes 2. No 3. I don’t know |  |
| 702 | If “yes’’ to question No 701, which type? You can answer more than one | 1. Pethidine 2. Diclofenac 3. Paracetamol 4. Hyoscine 5. If other specify________ |  |
| 703 | Have you got any special training for managing labour pain? | 1. Yes 2. No |  |
| 704 | Does your public center allow companion of her choice for laboring mother? | 1. Yes 2. No |  |
| 705 | Do you allow the women to move around while she is in labour? | 1. Yes 2. No |  |

THANK YOU
